# Long non-coding RNA ZFAS1 correlates with clinical progression and prognosis in cancer patients

**DOI:** 10.18632/oncotarget.18633

**Published:** 2017-06-27

**Authors:** Fangteng Liu, Hui Gao, Siyu Li, Xiaolin Ni, Zhengming Zhu

**Affiliations:** ^1^ Department of General Surgery, The Second Affiliated Hospital of Nanchang University, Nanchang 330000, Jiangxi Province, P. R. China; ^2^ The Second Clinical Medical College of Nanchang University, Nanchang 330000, Jiangxi Province, P. R. China

**Keywords:** ZFAS1, long non-coding RNA, cancer, prognosis, biomarker

## Abstract

Dysregulation of long non-coding RNA zinc finger antisense 1 (ZFAS1) has been reported in many types of cancers. We performed a synthetic analysis to clarify its prognostic significance as a cancer molecular-marker. Several databases (including PubMed, Web of Science, Embase together with Wanfang and China National Knowledge Internet database) were retrieved to identify ZFAS1-related articles. A total of eight articles were included in this meta-analysis. Hazard ratios (HR) and 95% confidence intervals (CI) were applied to assess the association between ZFAS1 expression level and overall survival (OS). Odds ratios (OR) were calculated with RevMan 5.3 software to determine the relationship between ZFAS1 expression and clinicopathologic features. The pooled results of the meta-analysis indicated that high ZFAS1 expression level was positively correlated with poor OS (HR = 1.87, 95% CI: 1.38-2.36, p< 0.001) in human solid cancers. The statistical significance was also observed in subgroup analysis stratified by the cancer type, analysis method, sample size and follow-up time. Furthermore, the elevated ZFAS1 expression was significantly related to positive lymph node metastasis (OR = 4.18, 95% CI: 2.70-6.48, p< 0.001). The present results suggest that ZFAS1 might be served as a novel promising biomarker for prognosis in Chinese patients with solid cancers.

## INTRODUCTION

Cancer is a leading cause of mortality worldwide with 14.1 million newly diagnosed cases and 8.2 million deaths each year [1, 2]. Albeit some dramatic therapeutic advances have been developed, survival rate in 5 years was still poor for the majority of cancer patients since malignant progression [2]. Hence novel biomarkers for predicting clinical prognosis are critical and necessary for prognostic accuracy and therapeutic decision-making.

Long noncoding RNA (lncRNA) are mRNA-like transcripts with more than 200 nucleotides in length and incapable of protein synthesis [3]. Initially it was considered as the “noise” of transcriptions, however, nowadays it has attracted great attentions for its major regulatory roles in diverse biological processes and diseases [4–5]. Dysregulation of lncRNAs has been reported in cancers and it was correlated with evolution, progression and prognosis in many kinds of cancers, they appear to be potential new prognostic biomarkers in cancers [6–7].

ZFAS1, short for zinc finger antisense 1, has been a newly identified lncRNA [8]. It was over-expressed in various kinds of tumors, such as breast cancer, ovarian cancer and gastric cancer [9–11]. Located on the antisense strand of the Znfx1 promoter region, lncRNA ZFAS1 suggested up-regulated tumorigenesis and advanced cancer progression. ZFAS1 played an important role in tumor biological activities, including cancer growth, proliferation and metastasis. The expression of ZFAS1 also reported to be positively associated with clinical outcome and it might be served as a promising prognostic marker in certain cancers [12–13]. However, owing to the discrete outcome and limited sample size provided by individual studies, there has been still no consensus on the prognostic value of ZFAS1 in cancer patients. Therefore, this meta-analysis was performed to comprehensively elaborate the prognostic value of ZFAS1 as a candidate biomarker for human cancer.

## RESULTS

### Literature retrieval and analysis

**Figure 1:**
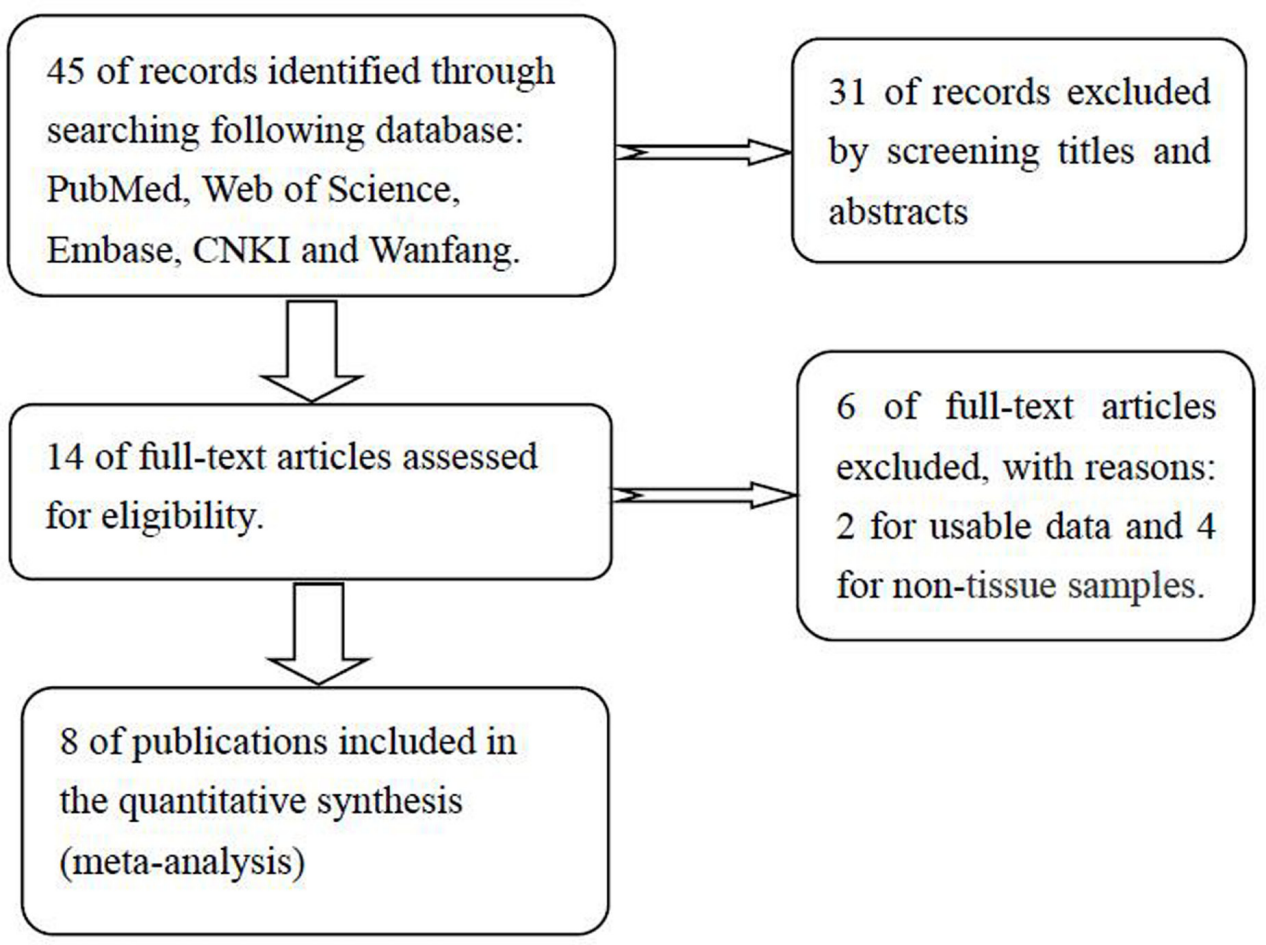
The flow diagram of this meta-analysis

A flow diagram of literature retrieval process was presented (Figure 1). Based on the standard of article selection, finally a total of 8 publications were included in this meta-analysis [12–19]. They have been all written in English and come from P. R. China. A total of 6 different types of cancer were included in present analysis, with 2 colorectal cancer, 2 gastric cancer, 1 hepatocellular carcinoma, 1 non-small cell lung cancer, 1 epithelial ovarian cancer and 1 glioma. The publication time of all included articles has been ranged from 2015 to 2017. The main characteristics were summarized (Table 1). The diagnosis of cancer was histopathologically confirmed. The expression level of lncRNA ZFAS1 was determined with qRT-PCR. There were three different cut-off points for the 8 included publications: 4 median level, 1 median ratio and 4 not reported. All included studies were assessed to be of high quality by the Newcastle-Ottawa Scale.

**Table 1:** The basic characteristics of all included studies in the meta-analysis

First author, year	Country	Cancer type	Samples	Tumor stage	Preoperative chemoradiotherapy	Cut-off value	Detection method	Outcome measure	Follow-upperiod	Analysis method
Li T, 2015 [12]	China	HCC	88	I-III	*NR*	Median level	qRT-PCR	OS	<5 years	Kaplan–Meier
Fang C, 2016 [13]	China	CRC	294	I-IV	*NR*	*NR*	qRT-PCR	OS	≥ 5 years	Kaplan–Meier
Nie F, 2016 [14]	China	GC	54	I-IV	None	Median level	qRT-PCR	OS	<5 years	Kaplan–Meier
Tian FM, 2016 [15]	China	NSCLC	173	I-IV	None	*NR*	qRT-PCR	OS	≥ 5 years	Multivariate analysis
Wang W, 2016 [16]	China	CRC	159	I-IV	*NR*	Median level	qRT-PCR	OS	≥ 5 years	Multivariate analysis
Zhang JJ, 2016 [17]	China	GC	104	I-IV	None	Median ratio	qRT-PCR	OS	≥ 5 years	Multivariate analysis
Gao K, 2017 [18]	China	Glioma	46	I-IV	None	*NR*	qRT-PCR	OS	<5 years	Kaplan–Meier
Xia B, 2017 [19]	China	EOC	60	I-IV	None	*NR*	qRT-PCR	OS	≥ 5 years	Kaplan–Meier

HCC: hepatocellular carcinoma; CRC: colorectal cancer; GC: gastric cancer; NSCLC: non-small cell lung cancer; EOC: epithelial ovarian cancer; qRT-PCR: quantitative reverse-transcriptase polymerase chain reaction; OS: overall survival; NR: not reported.

### Association between lncRNA ZFAS1 expression and OS

**Figure 2:**
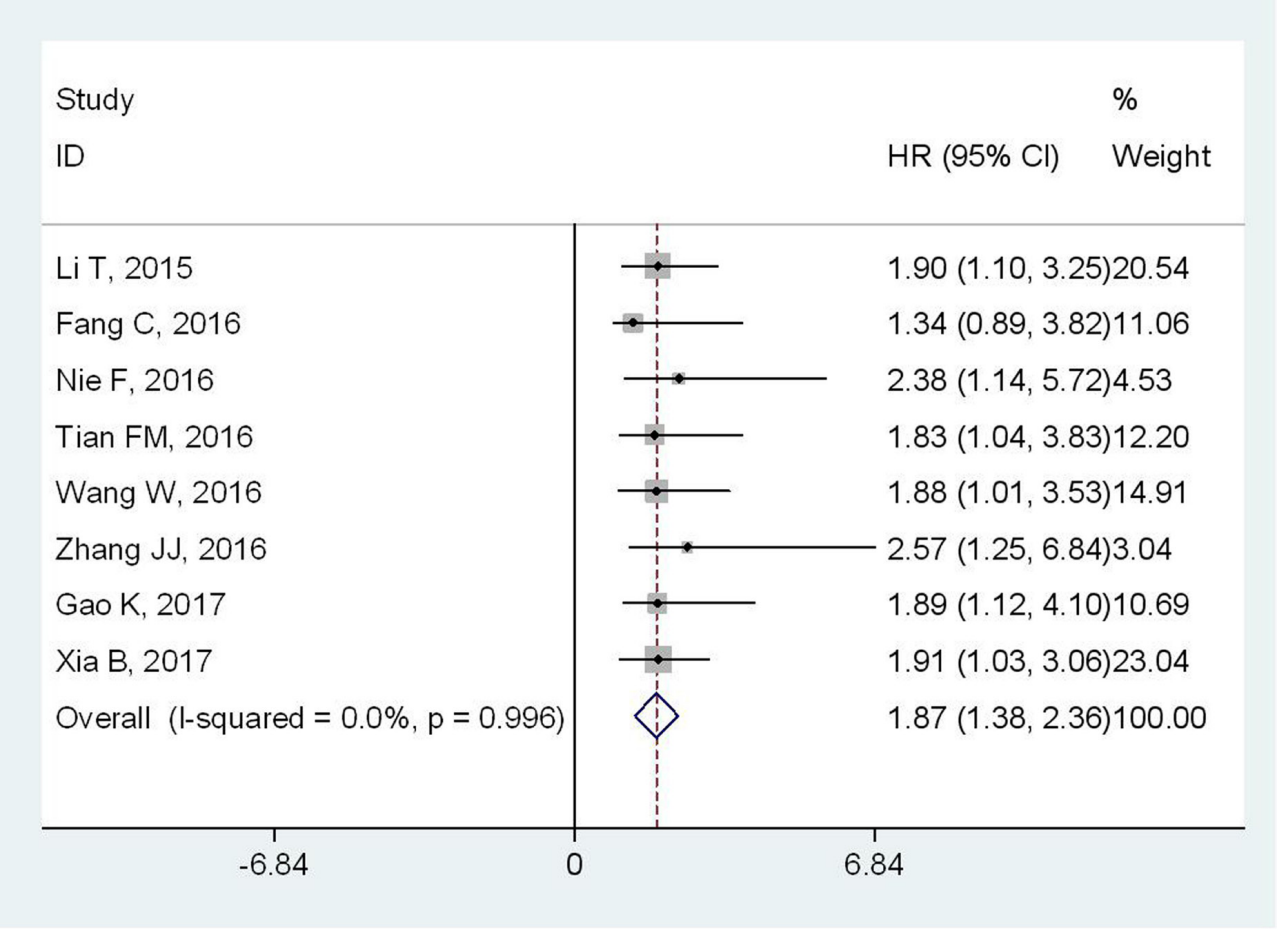
Forest plot showing the result of the pooled HRs of OS with overexpression of lncRNA ZFAS1 in different cancer types

All included articles investigated the association between ZFAS1 expression and OS with a total of 978 cancer patients. Because there was no significant heterogeneity among studies (I^2^=0.0%, P_Q_=0.919), the fixed-effects model was applied to calculate the pooled HR with corresponding 95% CI. Meta-analysis of those studies revealed that high ZFAS1 expression level was positively associated with poor OS in human cancer (HR: 1.87, 95% CI: 1.38-2.36, p< 0.001) (Figure 2).

Then the subgroup meta-analysis was performed, which was stratified by the cancer type, analysis method, sample size and follow-up time (Table 2). From the subgroup results, we found that high expression level of lncRNA ZFAS1 was significantly associated with shorter OS in patients with gastrointestinal cancer (HR: 1.86, 95% CI: 1.19-2.52, p< 0.001) and non-gastrointestinal cancer (HR: 1.88, 95% CI: 1.17–2.60, p< 0.001). Statistical significance was also found in subgroup analysis stratified by sample size, both equal or greater than 100 (HR:1.77, 95% CI: 1.01-2.53, p< 0.001) and less than 100(HR: 1.94, 95% CI: 1.30-2.57, p< 0.001). The association between ZFAS1 and OS of patients was significantly positively in studies reported in multivariate analysis (HR:1.93, 95% CI:1.04-2.82, p< 0.001) and non-multivariate analysis (HR: 1.84, 95% CI:1.26-2.43, p< 0.001). Additionally, a significant relationship between ZFAS1 expression level and OS of patients was observed in studies with follow-up times, both equal or greater than 5 years (HR: 1.82, 95% CI:1.21-2.43, p< 0.001) and less than 5 years (HR: 1.96, 95% CI: 1.14-2.77, p< 0.001).

**Table 2:** Results of subgroup analysis of pooled HRs of OS of patients with overexpression of lncRNA ZFAS1

Stratified analysis	No. of studies	No. of patients	Pooled HR (95% CI)	p-value	Heterogeneity		
					I^2^ (%)	P-value	Model
[1] Cancer type							
Gastrointestinal cancer	5	699	1.86 (1.19-2.52)	< 0.001	0.0	0.919	Fixed effects
Non-gastrointestinal cancer	3	279	1.88 (1.17-2.60)	< 0.001	0.0	0.996	Fixed effects
[2] Sample size							
≥ 100	4	730	1.77 (1.01-2.53)	< 0.001	0.0	0.877	Fixed effects
< 100	4	248	1.94 (1.30-2.57)	< 0.001	0.0	0.985	Fixed effects
[3] Analysis type							
Multivariate	3	436	1.93 (1.04-2.82)	< 0.001	0.0	0.891	Fixed effects
Non-multivariate	5	542	1.84 (1.26-2.43)	< 0.001	0.0	0.952	Fixed effects
[4] Follow-up time							
≥5 years	5	790	1.82 (1.21-2.43)	< 0.001	0.0	0.948	Fixed effects
<5 years	3	188	1.96 (1.14-2.77)	< 0.001	0.0	0.928	Fixed effects

### Association between lncRNA ZFAS1 expression and clinicopathological features

From the pooled results (Table 3), we found that overexpression of ZFAS1 was significantly associated with positive lymph node metastasis (OR = 4.18, 95% CI: 2.70-6.48, p<0.001) and advanced clinical stage (OR = 3.36, 95%CI: 1.69-6.65, p<0.001). However, the heterogeneity was observed in the association between overexpression of ZFAS1 and advanced clinical stage (I^2^= 77%). No significant associations were observed between high expression of ZFAS1 and gender (OR = 0.90, 95% CI: 0.66-1.22, p = 0.48) or tumor differentiation (OR = 1.54, 95% CI: 0.76-3.12, p = 0.23). Due to the insufficient data, we failed to explore the correlation between the overexpression of ZFAS1 and other clinicopathological features.

**Table 3:** Meta-analysis results of the associations of high lncRNA ZFAS1 expression level with clinicopathological features

Clinicopathological parameters	Studies (n)	Number of patients	OR (95% CI)	p-value	Heterogeneity		
					I^2^ (%)	P_Q_	Model
Gender (Male vs. Female)	6	676	0.90(0.66-1.22)	0.48	9	0.36	Fixed effects
Histological grade (Poorly/Undifferentiated vs. Well/Moderately)	4	459	1.54(0.76-3.12)	0.23	62	0.05	Random effects
Lymph node metastasis (Yes vs. No)	4	410	4.18(2.70-6.48)	< 0.001	0	0.70	Fixed effects
TNM stage (III-IV vs. I-II)	7	736	3.36(1.69-6.65)	< 0.001	77	0.0002	Random effects

### Publication bias

**Figure 3:**
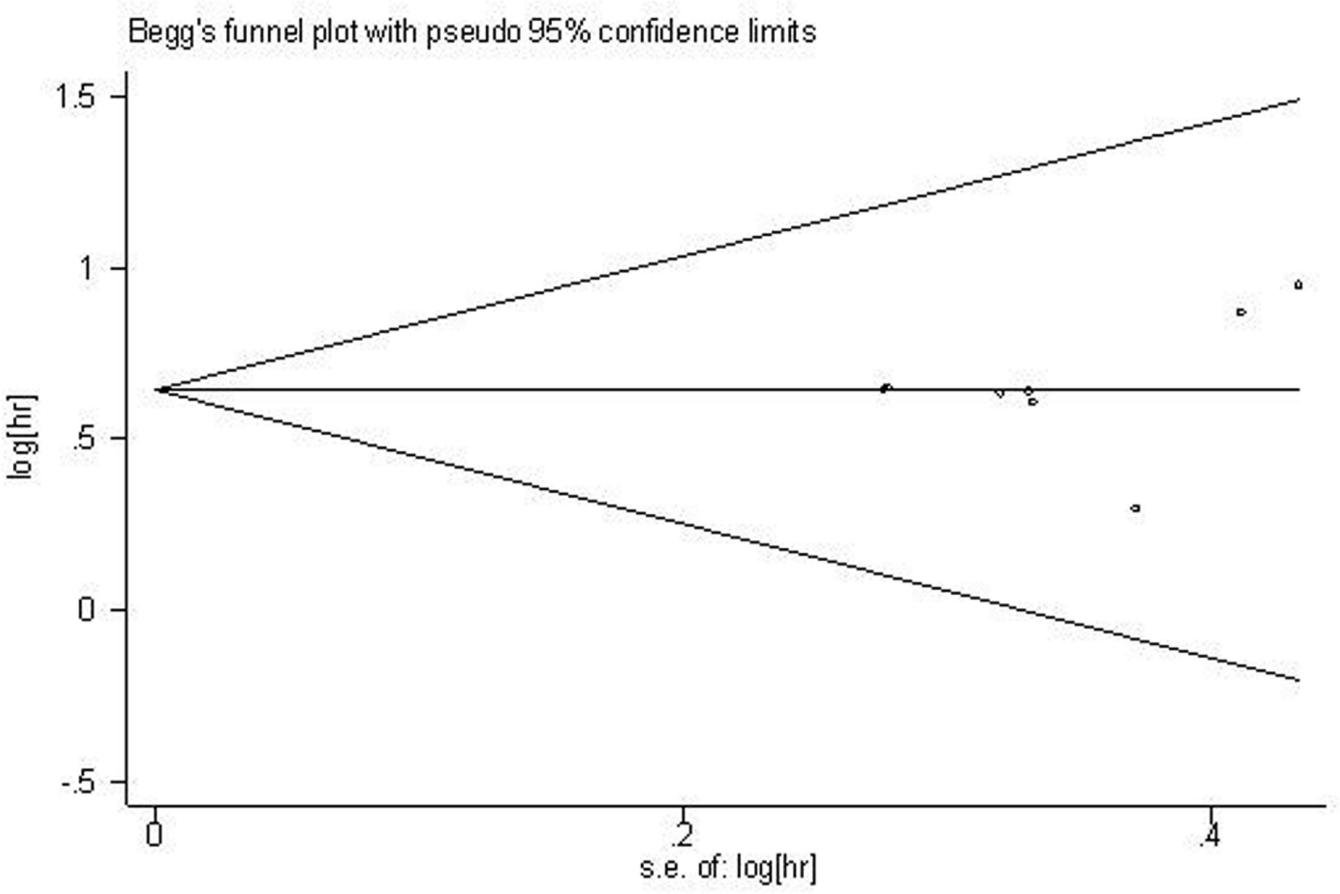
Funnel plot of the publication bias for the analysis of the pooled HRs of OS

For the meta-analysis of the association between ZFAS1 expression and OS, Begg’s funnel plot and Egger’s test results (*P* > |t| = 0.470, 95% CI: -1.985–3.811) showed there was no significant publication bias across the included studies (Figure 3).

### Sensitivity analysis

**Figure 4:**
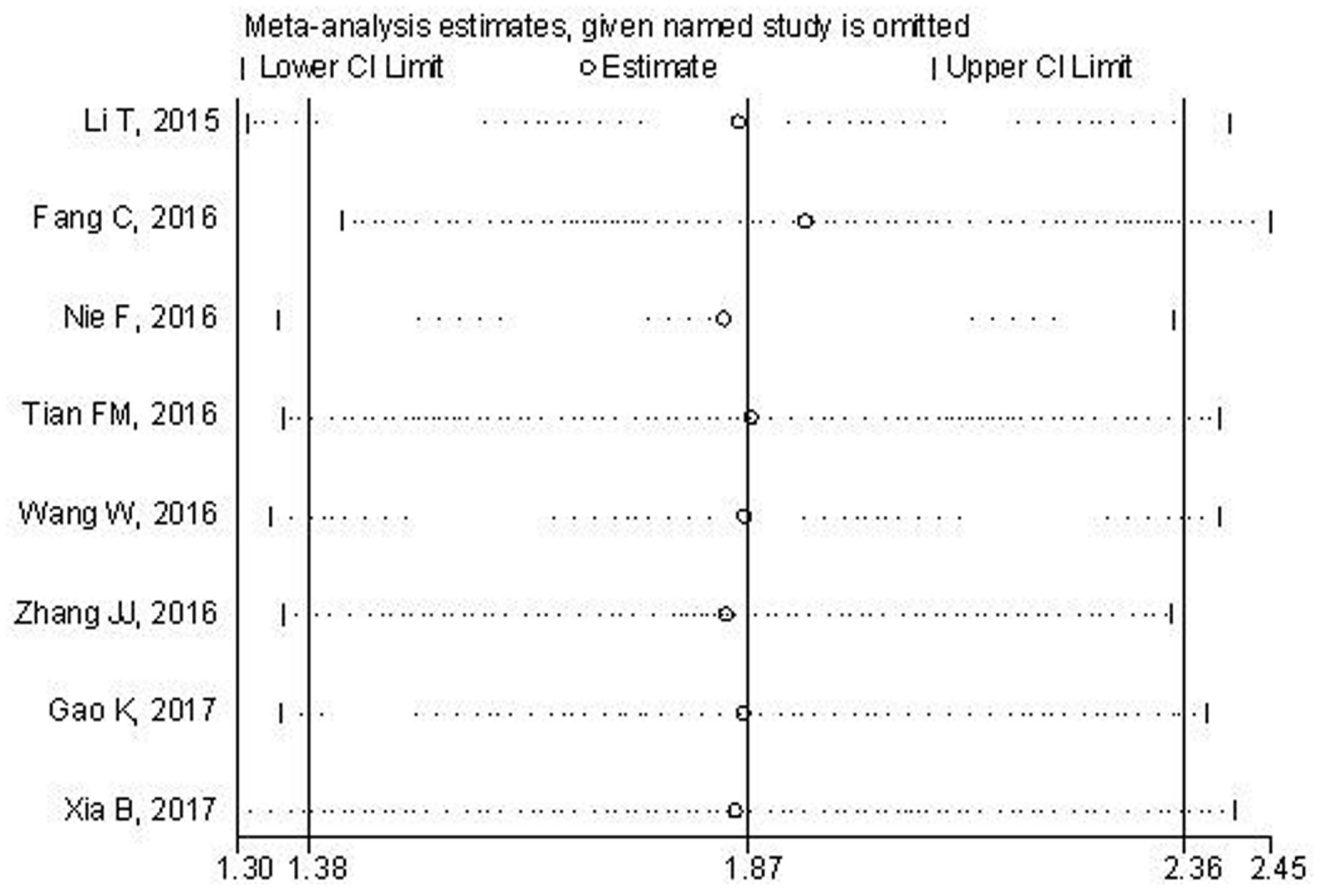
Sensitivity analysis of the pooled HRs of lncRNA ZFAS1 expression and OS

Sensitivity analysis was conducted to assess the effects of any single study on the overall outcome. After the exclusion of study, the pooled result was not significantly altered, suggesting the robustness of the results (Figure 4).

## DISCUSSION

Recently, increasing evidences revealed that lncRNAs play significant roles in various biological processes and various diseases [20–21]. Noteworthily, it found that lncRNAs were involved in tumorigenesis and tumor progression [22]. As emerging stars in cancer biomarkers, more and more lncRNAs were identified, and some functioned as oncogenes, such as CCAT2, HOTTIP and TUG1 [23–25], while some may be acted as tumor suppressor genes, such as MEG3, GAS5, ANRIL [26–28]. Aberrant expression of lncRNAs exerts impacts on the tumor biological processes and often correlated with aggressive progression and prognosis. Consequently, lncRNAs, as promising biomarkers for tumor prognosis and monitoring, have attracted great attentions [29–31].

LncRNA ZFAS1 is a newly identified lncRNA, which was originally reported by Askarian-Amiri et al. [8]. It reported that downregulation of ZFAS1 expression could promote cell proliferation, suggesting ZFAS1 as a putative tumor suppressor gene in breast cancer [8]. However, in some later researches, lncRNA ZFAS1 was reported to be up-regulated and regarded as an oncogene in tumorigenesis and progression. Although the roles of ZFAS1 in cancers are complex and still unclear, some research results have been obtained. In hepatocellular carcinoma (HCC), Li et al. [12] reported that ZFAS1 was frequently amplified in HCC and the upregulated expression of ZFAS1 could promote tumor metastasis in a miR-150-dependent manner and exert the tumor-promotive function. In colorectal cancer, lncRNA ZFAS1 may function as an oncogene by modulating ZEB1 to induce the expression of EMT [13], or combing with CDK1 and sponging with miR-590-3p to inhibit apoptosis [32]. It reported that ZFAS1 may be involved in the regulation of EMT in GC progression [11] and also could promote GC cells proliferation by epigenetically repressing KLF2 and NKD2 expression [14]. Additionally, ZFAS1 could exhibit a tumor oncogenic role by regulating EMT and Notch signaling pathway in glioma [18] and it facilitated ovarian cancer cell malignancy by participating in miR-150-5p/Sp1 axis [19].

Regarding to the clinical value of ZFAS1 expression in human cancers, Zhang et al. [17] showed that high expression of ZFAS1 was correlated with deeper depth of invasion, LNM and higher TNM stage in GC patients. And Wang et al. [16] reported that elevated ZFAS1 expression in CRC was correlated with lymphatic invasion and advanced TNM stage and its expression was associated with relapse and survival. Similarly, Fang et al. [13] found that there was significant correlation between ZFAS1 expression and LNM, TNM stage but not gender or differentiation, ZFAS1 upregulation was also correlated with poor prognosis in colonic cancer. However, in NSCLC, Tian et al [15] showed that there was a significant association between ZFAS1 expression and differentiation. Thus it can be seen that the prognostic value of ZFAS1 in cancer patients were inconclusive or even contradictory, the role of ZFAS1 as a tumor biomarker for prognosis was unclear. Thus, our study aimed to assess the prognostic value of ZFAS1 in cancer patients via a meta-analysis.

Our meta-analysis showed that overexpression of ZFAS1 was correlated with unfavorable clinical outcomes in cancer patients. Compared to those with low expression, the patients with high ZFAS1 expression have a shorter OS (HR: 1.87, 95% CI: 1.38-2.36). And subgroup analyses were also performed to assess the prognostic value of ZFAS1, the results indicated that high expression level of ZFAS1 might be an independent prognostic factor for OS. Furthermore, we explored the relationship between ZFAS1 expression and pathological features. We found that there was a significant association between ZFAS1 expression and LNM (OR=4.18; 95% CI: 2.70-6.48) and clinical stage (OR = 3.36, 95%CI: 1.69-6.65). However, the heterogeneity was observed in the association between ZFAS1 expression and clinical stage, thus, the results should be further confirmed. Taken together, ZFAS1 was involved in tumor development and progression and may act as a potential predictive factor for clinical outcomes.

Nevertheless, some limitations should be taken into account when interpreting our conclusions. First, the total numbers of cancer patients and included cancer types were relative small. Second, the cut-off value of ZFAS1 expression in included studies was not all the same. Third, all studies were from China and this might limit the applicability of our findings for other ethnic groups. Fourth, some studies included provided Kaplan-Meier curves for estimating the HR and 95%CIs, this might influence the accuracy of results. Fifth, positive results published more easily than negative results. Additionally, studies are needed to confirm the specific functions of ZFAS1 in different kinds of cancers.

In conclusion, upregulation of lncRNA ZFAS1 was associated with higher positive rate of LNM, more advanced clinical stage and poorer OS in cancer patients. Our results suggested that lncRNA ZFAS1 might serve as a novel molecular marker for clinical progression and prognosis for cancer patients in China. In the future, we still need further well-designed studies with larger sample and other ethnic groups to confirm its prognostic significance in cancers.

## MATERIALS AND METHODS

### Literature retrieval to identify relevant articles for meta-analysis

Relevant studies regarding to the prognostic significance of lncRNA ZFAS1 in human cancers were comprehensively searched via the following online databases: PubMed, Web of Science, Embase together with Wanfang and China National Knowledge Internet (CNKI) databases. Both free-text words and MeSH terminology were applied to maximize chances of securing the eligible articles. The searching terms were: “Zinc finger antisense 1”, “ZFAS1”, “lncRNA ZFAS1” and “long noncoding RNA ZFAS1”. The latest search was updated on February 10, 2017. Manual searches were also performed in the reference lists of primary articles. The full-text articles written in both English and Chinese were included in this meta-analysis.

### Selection criteria for study inclusion

Inclusion criteria included: (1) Studies exploring the association between lncRNA ZFAS1 expression and human cancer patients; (2) The expression level of lncRNA ZFAS1 in tissue specimens was determined; (3) The association between lncRNA ZFAS1 expression and overall survival were described; (4) Sufficient data were provided for calculating the hazard ratio (HR) with 95% CI for survival rates.

Exclusion criteria included: (1) duplicate publications; (2) reviews, case reports and conference abstracts; (3) studies without available data.

### Data extraction and quality assessment

Through a same standardized information collection forms, following details were extracted by two investigators independently (LSY and NXL): first author’s name, publication year, country of origin, cancer type, sample size, tumor stage, follow-up period, detected methods, the cut-off value, and HR as well as corresponding 95% CI. Additionally, the clinicopathological features (gender, histopathological grade, lymph node metastasis and TNM stage) were also extracted from all included studies. Any disagreements were consulted with a third investigator.

For the survival data extraction, the data was directly applied if a study reported the detailed HRs and 95% CIs for survival. Otherwise, the HRs and 95% CIs was retrieved with Engauge Digitizer version 4.1(http://digitizer.sourceforge.net/) if a study only provide Kaplan-Meier curves. Any essential information not available from the original article were obtained by sending emails to contact the corresponding authors.

For the quality assessment, it was performed with Newcastle-Ottawa quality assessment scale (NOS) since all included studies were non-randomized studies. There was a score ranging from 0 to 9 points in the method. A study with a score ≥ 6 was regarded as high quality, suggesting lower risks of bias. In this meta-analysis, the quality scores of all included studies were varied from 6 to 9, with a mean value of 7.5.

### Statistical analysis

This present meta-analysis was performed with Stata statistical software version 12.0 (*Stata Corporation, College Station, Texas, USA*) and RevMan5.3 software (Cochrane Collaboration, http://www.cc-ims.net/RevMan/relnotes.htm/).

The heterogeneity among studies was identified via I^2^ statistics and chi-square Q test. If there was significant heterogeneity between-studies (I^2^ ≥ 50% and P_Q_ ≤ 0.05), the random effects model was adopted; When heterogeneity was not significant (I^2^ < 50% and P_Q_ > 0.05), the fixed effects model was used. Subgroup analysis was performed to further assess the prognostic value of lncRNA ZFAS1 [33]. Sensitivity analysis were applied to evaluate the stability of the results. The publication bias was assessed with the Begg’s funnel plot and Egger’s test. Statistical significance was defined when the p-value was less than 0.05.

## References

[1] Ferlay J, Soerjomataram I, Dikshit R, Eser S, Mathers C, Rebelo M, Parkin DM, Forman D, Bray F (2015). Cancer incidence and mortality worldwide: sources, methods and major patterns in GLOBOCAN 2012. Int J Cancer.

[2] Siegel RL, Miller KD, Jemal A (2016). Cancer statistics, 2016. CA Cancer J Clin.

[3] Mattick JS (2009). The genetic signatures of noncoding RNAs. PLoS Genet.

[4] Deniz E, Erman B (2017). Long noncoding RNA (lincRNA), a new paradigm in gene expression control. Funct Integr Genomics.

[5] Beermann J, Piccoli MT, Viereck J, Thum T (2016). Non-coding RNAs in development and disease: background, mechanisms, and therapeutic approaches. Physiol Rev.

[6] Tano K, Akimitsu N (2012). Long non-coding RNAs in cancer progression. Front Genet.

[7] Jiang C, Li X, Zhao H, Liu H (2016). Long non-coding RNAs: potential new biomarkers for predicting tumor invasion and metastasis. Mol Cancer.

[8] Askarian-Amiri ME, Crawford J, French JD, Smart CE, Smith MA, Clark MB, Ru K, Mercer TR, Thompson ER, Lakhani SR, Vargas AC, Campbell IG, Brown MA (2011). SNORD-host RNA Zfas1 is a regulator of mammary development and a potential marker for breast cancer. RNA.

[9] Zhang Z, Weaver DL, Olsen D, deKay J, Peng Z, Ashikaga T, Evans MF (2016). Long non-coding RNA chromogenic *in situ* hybridisation signal pattern correlation with breast tumour pathology. J Clin Pathol.

[10] Liu R, Zeng Y, Zhou CF, Wang Y, Li X, Liu ZQ, Chen XP, Zhang W, Zhou HH (2017). Long noncoding RNA expression signature to predict platinum-based chemotherapeutic sensitivity of ovarian cancer patients. Sci Rep.

[11] Zhou H, Wang F, Chen H, Tan Q, Qiu S, Chen S, Jing W, Yu M, Liang C, Ye S, Tu J (2016). Increased expression of long noncoding RNA ZFAS1 is associatedwith epithelial-mesenchymal transition of gastric cancer. Aging (Albany NY).

[12] Li T, Xie J, Shen C, Cheng D, Shi Y, Wu Z, Deng X, Chen H, Shen B, Peng C, Li H, Zhan Q, Zhu Z (2015). Amplification of long noncoding RNA ZFAS1 promotes metastasis in hepatocellular carcinoma. Cancer Res.

[13] Fang C, Zan J, Yue B, Liu C, He C, Yan D (2017). Long noncoding RNA ZFAS1 promotes the progression of colonic cancer by modulating ZEB1 expression. J Gastroenterol Hepatol.

[14] Nie F, Yu X, Huang M, Wang Y, Xie M, Ma H, Wang Z, De W, Sun M (2017). Long noncoding RNA ZFAS1 promotes gastric cancer cells proliferation by epigenetically repressing KLF2 and NKD2 expression. Oncotarget.

[15] Tian FM, Meng FQ, Wang XB (2016). Overexpression of long-noncoding RNA ZFAS1 decreases survival in human NSCLC patients. Eur Rev Med Pharmacol Sci.

[16] Wang W, Xing C (2016). Upregulation of long noncoding RNA ZFAS1 predicts poor prognosis and prompts invasion and metastasis in colorectal cancer. Pathol Res Pract.

[17] Zhang JJ, Chen JT, Yao KH, Hua L, Wang CY, Hu JH (2016). Up-regulated expression of long non-coding RNA ZFAS1 associates with aggressive tumor progression and poor prognosis in gastric cancer patients. Int J Clin Exp Pathol.

[18] Gao K, Ji Z, She K, Yang Q, Shao L (2017). Long non-coding RNA ZFAS1 is an unfavourable prognostic factor and promotes glioma cell progression by activation of the Notch signaling pathway. Biomed Pharmacother.

[19] Xia B, Hou Y, Chen H, Yang S, Liu T, Lin M, Lou G (2017). Long non-coding RNA ZFAS1 interacts with miR-150-5p to regulate Sp1 expression and ovarian cancer cell malignancy. Oncotarget.

[20] Liu Y, Zheng L, Wang Q, Hu YW (2017). Emerging roles and mechanisms of long noncoding RNAs in atherosclerosis. Int J Cardiol.

[21] Lavorgna G, Vago R, Sarmini M, Montorsi F, Salonia A, Bellone M (2016). Long non-coding RNAs as novel therapeutic targets in cancer. Pharmacol Res.

[22] Li J, Tian H, Yang J, Gong Z (2016). Long noncoding RNAs regulate cell growth, proliferation, and apoptosis. DNA Cell Biol.

[23] Xin Y, Li Z, Zheng H, Chan MT, Ka Kei Wu W (2017). CCAT2: a novel oncogenic long non-coding RNA in human cancers. Cell Prolif.

[24] Lian Y, Cai Z, Gong H, Xue S, Wu D, Wang K (2016). HOTTIP: a critical oncogenic long non-codingRNA in human cancers. Mol Biosyst.

[25] Li Z, Shen J, Chan MT, Wu WK (2016). TUG1: a pivotal oncogenic long non-coding RNA of human cancers. Cell Prolif.

[26] Zhou Y, Zhang X, Klibanski A (2012). MEG3 noncoding RNA: a tumor suppressor. J Mol Endocrinol.

[27] Ma C, Shi X, Zhu Q, Li Q, Liu Y, Yao Y, Song Y (2016). The growth arrest-specific transcript 5 (GAS5): a pivotal tumor suppressor long noncoding RNA in human cancers. Tumour Biol.

[28] Li Z, Yu X, Shen J (2016). ANRIL: a pivotal tumor suppressor long non-coding RNA in human cancers. Tumour Biol.

[29] Liu FT, Pan H, Xia GF, Qiu C, Zhu ZM (2016). Prognostic and clinicopathological significance of long noncoding RNA H19 overexpression in human solid tumors: evidence from a meta-analysis. Oncotarget.

[30] Liu FT, Zhu PQ, Luo HL, Zhang Y, Qiu C (2016). Prognostic value of long non-coding RNA UCA1 in human solid tumors. Oncotarget.

[31] Liu FT, Xue QZ, Zhu PQ, Luo HL, Zhang Y, Hao T (2016). Long noncoding RNA AFAP1-AS1, a potential novel biomarker to predict the clinical outcome of cancer patients: a meta-analysis. Onco Targets Ther.

[32] Thorenoor N, Faltejskova-Vychytilova P, Hombach S, Mlcochova J, Kretz M, Svoboda M, Slaby O (2016). Long non-coding RNA ZFAS1 interacts with CDK1 and is involved in p53-dependent cell cycle control and apoptosis in colorectal cancer. Oncotarget.

[33] Hogeveen M, Blom HJ, den Heijer M (2012). Maternal homocysteine and small-for-gestational-age offspring: systematic review and meta-analysis. Am J Clin Nutr.

